# A Compact Dual-Band Notched UWB Antenna for Wireless Applications

**DOI:** 10.3390/mi13010012

**Published:** 2021-12-23

**Authors:** Om Prakash Kumar, Pramod Kumar, Tanweer Ali

**Affiliations:** Department of Electronics and Communication Engineering, Manipal Institute of Technology, Manipal Academy of Higher Education, Manipal 576104, India; omprakash.kumar@manipal.edu

**Keywords:** U and V-shaped slot, UWB, parasitic resonator, notch

## Abstract

This article presents the design and analysis of a V-shaped ultrawideband (UWB) antenna and dual-band UWB notch antenna. A rectangular slot is cut into a semicircular partial ground plane of the antenna to achieve ultrawide bandwidth. A U-shape slot is etched on a V-shaped patch that radiates, and an inverted U-shape parasitic resonator is placed beside the feedline to generate dual-band notch characteristics. The overall dimension of the proposed antenna is $28\times 23{\;\mathrm{mm}}^{2}$. The proposed UWB antenna has a gain of 9.8 dB, S_11_ < −10 dB, impedance bandwidth in the range of 3.4 to 12.3 GHz, response with a linear phase, group delay <1 ns, and stable radiation pattern. The UWB notch antenna shows strong rejection in the WLAN band from 5.15 to 5.8 GHz with a notch at 5.6 GHz and X band from 9.1 to 10.5 GHz with a sharp notch at 9.6 GHz, having a S_11_ < −10 dB impedance bandwidth ranging from 3.2 to 11.7 GHz. This antenna also exhibits a stable radiation pattern, group delay <1 ns, and linear phase response throughout the bandwidth except at the rejection frequencies.

## 1. Introduction

With the advancement in technology through intensive research and development, the wireless communication system has leapt forward in particular compared to other systems. This development in wireless communication resulted in forming a regulatory body, the Federal Communication Commission (FCC), to serve broadband, radiofrequency, and public safety use [1].

### 1.1. UWB Antenna

The Federal Communications Commission (FCC) describes ultrawideband (UWB) as systems that comprise a bandwidth higher than 500 MHz. In terms of fractional bandwidth, values higher than 120% are considered to be a part of UWB systems. A bandwidth of around 7.5 GHz has been allotted for UWB operation, exhibiting a power emission of −41.3 dBm/MHz [2]. UWB systems hold a plethora of applications such as imaging, communications, radar signaling, positioning, etc. as it promises features viz., improved rate of data transmission and reception, large bandwidth, reduced power consumption, and cost-effectiveness [3]. The channel capacity associated with its frequency bandwidth can handle a high date rate efficiently. Since the UWB pulse duration is small, it is easier to get a high data rate. A high data rate enables wireless communication to work with low latency and provides error-proof data transfer [4]. The antenna has a significant effect on UWB communication systems performance. Therefore, an antenna design must meet certain standard specifications, viz., impedance matching, stable radiation pattern, small size, and cost effectiveness, which is considerably challenging. The microstrip patch antennas are suitable candidates for UWB utilization because they are light in weight, possess a planar geometry, and can easily be integrated with other electronic elements [5,6].

In this view, several UWB antennas that are compact have been showcased in the literature [7,8,9,10,11,12,13,14]. Kundu et al. [7] recommended a CPW fed UWB antenna shaped like a leaf, which operates in the 2.58–11.62 GHz range. Krishna et al. [8] proposed a dual-polarized UWB antenna with slots. The matching of impedance is achieved using identically shaped slots and rectangular stepping. Similarly, Alsath et al. [9] used half-circular and square ring geometry to create a UWB antenna with increased bandwidth for applications in the automotive sector with a bandwidth of 3.1 to 10.94 GHz. Ali et al. [10] proposed a UWB antenna in the shape of a Volkswagen with a bandwidth of 3.1 to 11.8 GHz. Li et al. [11] have designed a slot antenna with low dispersion characteristics having a good gain and an overall dimension of 106 × 68 mm^2^ with an impedance bandwidth of 1.4 to 3.5 GHz. Mohammad et al. [12] designed a UWB antenna with a frequency of operation between 2.8 and 12.2 GHz using slots in the shape of concentric rings for size reduction of the the antenna. In the paper [13], the authors have proposed a compact UWB antenna, which comprises a circular patch and partial ground plane with a rectangular slot on the top. The antenna operates from 3.3 to 20 GHz. It shows a maximum gain of 7.5 dBi and a stable radiation pattern. The antenna structure is simple and easy to fabricate. Using an annular ring patch and partial ground plane with a rectangular slot in [14], a microstrip-fed UWB monopole antenna was designed. The antenna exhibits a maximum gain of 5.5 dBi and a stable radiation pattern in the operating bandwidth from 2.6 to 12.3 GHz.

Notwithstanding the UWB antenna design improvements showcased in the literature above, there exist constraints for UWB antenna designs used in mobile terminals, especially for achieving acceptable values of gain and good impedance bandwidth. The antenna proposed in this article incorporates significant features for meeting the current requirements of a feasible UWB system.

### 1.2. UWB Notch Antenna

UWB systems present the greatest challenge of overcoming interference with narrowband systems such as WiMAX (3.3 to 3.7 GHz), WLAN (5 to 6 GHz), and X-band ITU (8.025 to 8.4 GHz). Therefore, it is crucial to minimize the interference at these frequency bands by involving band rejection techniques [15]. Notch antennas have been known for compact designs and have originally been used in missiles and aircraft flying surfaces. To decrease the interference due to narrowband systems, UWB systems make use of filters to selectively remove interfering frequency components. However, the usage of filters in the design of the UWB system enhances the complexity and thereby the cost of the system. The band-notched UWB systems find their applications in Personal Communications Systems (PCS) and other handheld devices that need to be compact [16].

Various band rejection techniques have been employed to analyze the performance of antennae by researchers [17]. Some of the technique involves etching slots in a T-shape on the patch or from the substrate by making use of U-shape [18], C-shape [19], and arc shape slots, which are some of the common slotting techniques. The demerits of making too many slots are that it makes the structure complex, with low gain and low impedance bandwidth. Another method involves installing open-ended stubs or placing parasitic patches on the radiating patch [20]. The demerits involving stubs and parasitic resonators reduce the gain and can cause a short circuit.

In this view, several single [21,22], dual [23,24], triple [25,26,27], quad [28,29], quintuple [30,31], and sextuple notch [32,33] UWB antennas have been showcased in the literature [21,22,23,24,25,26,27,28,29,30,31,32,33]. In [21], the authors have proposed a single-band notch antenna. A half-wave resonant structure in the patch is used to obtain a notch band at 5.6 GHz for band rejection of WLAN. In [22], the authors have proposed a single-band notch antenna. A rectangular slot in the ground plane is used to obtain a notch band of 3–4.7 GHz. In [23], the authors have proposed a dual-band notch antenna. A meandered slot in the patch and U-shape slot in the feed are used to obtain a notch band of 3–3.9 and 5.22–5.7 GHz. In [24], the authors proposed a dual-band UWB notch antenna. Two U-shaped stubs on the patch are used to obtain a notch band of 3.0–3.9 GHz and 5.0–5.9 GHz. A three-band notch antenna has been proposed in [25]. In this design, two bevels in the patch and two bevels in the ground are used to get a wide bandwidth. The usage of two round slots of half-wavelength along with two slots of C shape in the ground plane gives notches of3.3–3.7 GHz, 5.1–5.8 GHz, and 7.1–7.7 GHz. Similarly, a triple band-notched UWB antenna has been proposed in [26]. This design uses a split ring resonator (SRR) in the ground to notch WiMAX (3.3–3.8 GHz) and WLAN (5.15–5.825 GHz). The U-shape slot usage in the feed line gives a notched band of X-band (7.25–8.395 GHz). In [27], a UWB notch antenna was designed for 5G, WLAN, and Satellite downlink bands applications. The authors use electromagnetic bandgap (EBG) structures to notch 5G and WLAN bands and two split-ring resonators (SRR) to notch Satellite downlink bands. A four-band notch elliptical UWB antenna has been proposed in [28]. In this design, three slots of inverted U-shape and one I-shape slot are used to achieve a quadruple notch of 3.2–3.9 GHz, 4.3–5.0 GHz, 5.5–6.6 GHz, and 7.9–9.3 GHz.

A quadruple-notch UWB antenna has been proposed in [29]. In this design, a C-shaped slot in the patch and U-shaped slot in the feed achieve four notches of 2.44–2.77 GHz, 3.42–3.97 GHz, 5.45–5.98 GHz, and 8–8.68 GHz. A quintuple notch UWB antenna has been proposed in [30]. In this design, a Y-shaped antenna with an inverted U-shape slot along with a C-shape slot on the radiating patch and ground plane are utilized to achieve a quintuple notch of 3.4–4.0 GHz, 5.1–5.9 GHz, 6.7–8.0 GHz, 8.3–9.1 GHz, and 9.3–10.6 GHz. A quintuple-notch UWB antenna has been proposed in [31]. In this design, a Spiral line EBG and fork-shaped slot achieve a quintuple notch of 2.4–2.9 GHz, 3.6–4.3 GHz, 5.3–5.7 GHz, 6.4–6.7 GHz, and 7.8–8.6 GHz. A sextuple-notch UWB antenna has been proposed in [32]. In this design, an antenna with an elliptic radiator and a rectangle ground plane using ESRR and RSRR with a U-shaped parasitic strip is utilized to achieve a sextuple notch of 2.9–3.3 GHz, 3.7–3.8 GHz, 4.4–4.5 GHz, 5.3–5.5 GHz, 7.0–7.3 GHz, and 7.5–8.0 GHz. Similarly, another sextuple-notch UWB antenna has been proposed in [33]. In this, a stub with an open end with a meandered resonator along with a defective ground structure is used to achieve a sextuple notch of 3.3–3.6 GHz, 5.1–5.3 GHz, 5.6–5.9 GHz, 7.2–7.6 GHz, 7.8–8.2 GHz, and 9.2–9.7 GHz. However, for the present paper, the analysis of the proposed design is limited to a dual-notch band of operation, and its relevance is judged based on the antenna proposed in [21,22,23,24].

In this paper, a UWB antenna having a dual notch has been designed with the bandwidth of UWB (3.GHz to 12.3 GHz) and notched frequencies of WLAN at 5.6 GHz (5.15 GHz to 5.6 GHz) and X-band at 9.6 GHz (9.13 GHz to 10.5 GHz). U-shaped slots have been etched from the radiating patch, and a parasitic U-shape resonator is placed on the semicircular ground plane close to the feed line. The antenna is designed and simulated on HFSS. The fabricated UWB and UWB notch antenna approves the simulated results. The remaining section describes the antenna design approach, parametric analysis, time-domain analysis, and radiation pattern.

## 2. UWB Antenna Design Approach

**Figure 1:**
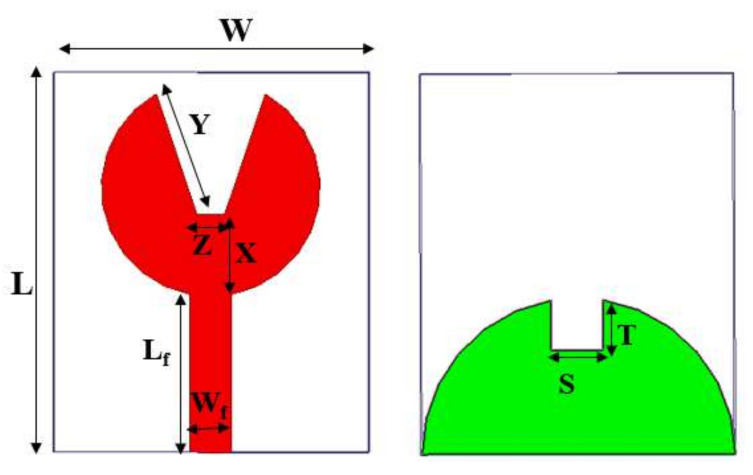
Proposed UWB antenna design.

The proposed V-shaped monopole UWB patch antenna is depicted in Figure 1. It has a size of $28\times 23\times 1.6{\;\mathrm{mm}}^{3}$. It consists of a radiating patch shaped as a circle with V-shape slots on the top and a semicircular ground plane with rectangular truncation on the bottom. The impedance matching of the feedline $\left(W_{f}\times L_{f}\right)$ is 50 Ω. The antenna is compacted by cutting slots in the patch for radiation and truncating the ground plane. Slots in the design change the current distribution and affect the inductance and capacitance, thereby making the antenna exhibit wide bandwidth. The advantage of our design is its simplicity and planar structure. The final antenna is simulated using HFSS software. The design dimensions are given in Table 1.

The resonance frequency of the V-shaped radiating patch can be calculated using Equations (1)–(3) given below:

(1) $$f_{r@4.1\;\mathrm{GHz}}=\frac{c}{4\times Y\times \sqrt{\varepsilon_{eff}}}$$

(2) $$f_{r@4.1\;\mathrm{GHz}}=\frac{3\times 10^{11}}{4\times 9.42\times \sqrt{\varepsilon_{eff}}}$$

(3) $$\mathrm{and},\;\varepsilon_{eff}=\frac{\varepsilon_{r}+1}{2}+\frac{\varepsilon_{r}-1}{2}\;\sqrt{\left(1+\frac{12h}{W}\right)}=3.74.$$

Putting the value of Equation (3) in Equation (2) we get

$$f_{r@4.1\;\mathrm{GHz}}=\frac{3\times 10^{11}}{4\times 9.42\times \sqrt{3.74}}=4.1\;\mathrm{GHz}$$

where *c* represents the light’s speed and is taken as $3\times 10^{11}$ mm/sec, *Y* is the maximum slotted patch current path, $\varepsilon_{eff}$ is the effective dielectric constant, *h* represents the thickness of the substrate, and *W* represents the substrate’s width.

### 2.1. Evolution Stage of the Antenna

**Figure 2:**
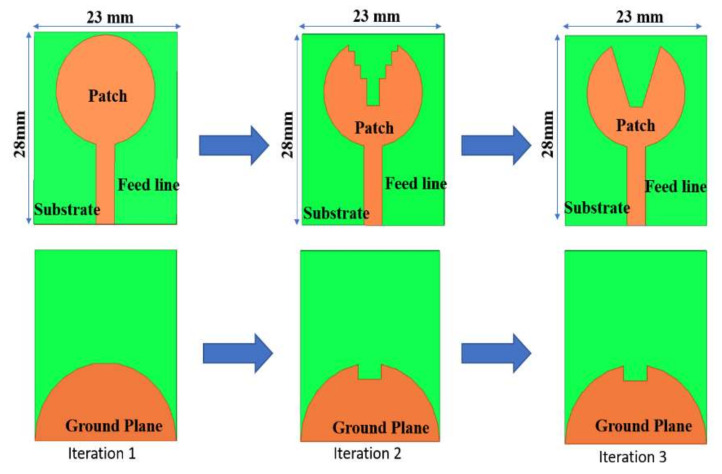
Evolution of the proposed design.

The proposed antenna structure is obtained by a series of evolution processes represented in Figure 2. In iteration 1, the antenna has a full circular radiating patch with a semicircular ground plane, which results in a narrow −10 dB impedance bandwidth from 3.4 to 4.8 GHz. There exists very poor impedance matching. To make a UWB, the impedance bandwidth is to be made wide. In iteration 2, Staircase slots are introduced on the circular radiating patch. A slot of rectangular shape is truncated on the semicircular ground plane. This is shown in iteration 2 of Figure 2. Compared to iteration 1, the impedance bandwidth is 3.5 to 12.3 GHz, but impedance matching is very poor, since the reflection coefficient curve touches a −10 dB line at 6 GHz frequency. Therefore, further modification is done in iteration 3 of Figure 2 to achieve UWB characteristics in the antenna. The staircase slots on the radiating patch are now cut into V-shape slots alongside keeping the truncation on the semicircular ground plane constant.

**Figure 3:**
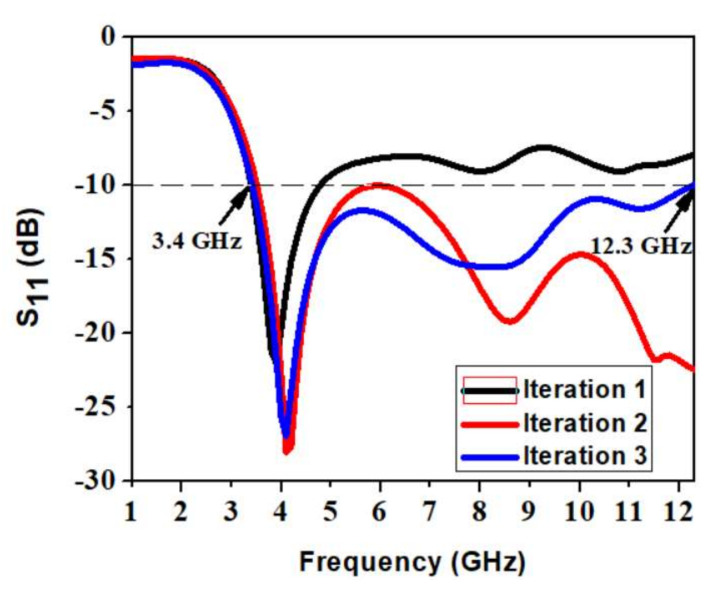
Variation in reflection coefficient with frequency for different antenna iterations.

It is now evident that the S11 < −10 dB bandwidth is 3.4 to 12.3 GHz, exhibiting ultrawide bandwidth. It has good impedance matching. Hence, the antenna has a resonating mode at 4.1 GHz and FBW of 113%, covering the entire UWB spectrum. Figure 3 illustrates the reflection coefficient of the evolutionary steps in the antenna design.

### 2.2. Parametric Analysis

A parametric analysis analyzes the patch element’s impact, the slots etched in the patch, and the antenna’s ground plane. The design’s performance is mainly influenced by the dimensions X, T, feed width, and ground plane structure.

#### 2.2.1. Effect of Variation of X

**Figure 4:**
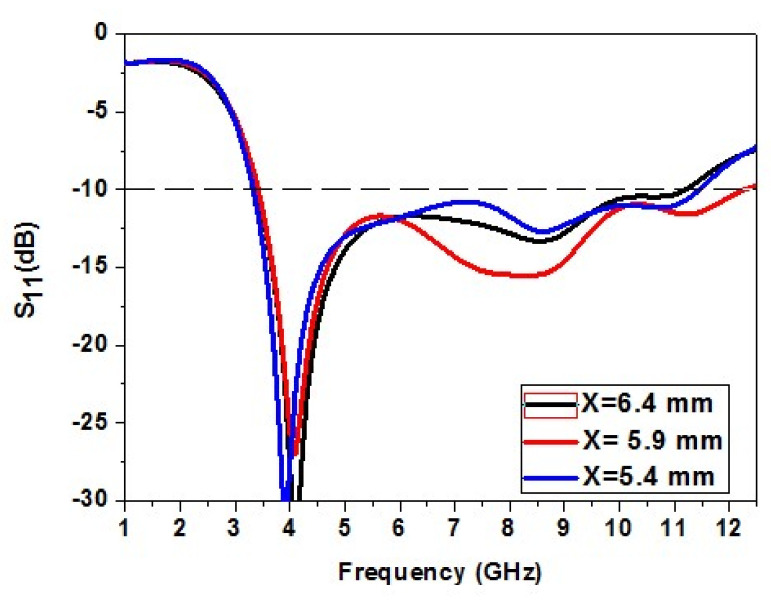
Analysis of variation in the dimension X of the patch element.

The effect of dimension X of the patch element on the antenna is studied. The dimension X of the element is increased and decreased to analyze its effect. The increase in X causes a decrease in impedance bandwidth. The reduction in X causes a slight rise in impedance bandwidth but is not as accepted. The dimension X = 5.9 mm gives the correct bandwidth. So, X = 5.9 mm is an optimized value. The variation of dimension X of the patch is shown in Figure 4.

#### 2.2.2. Effect of Variation of T

**Figure 5:**
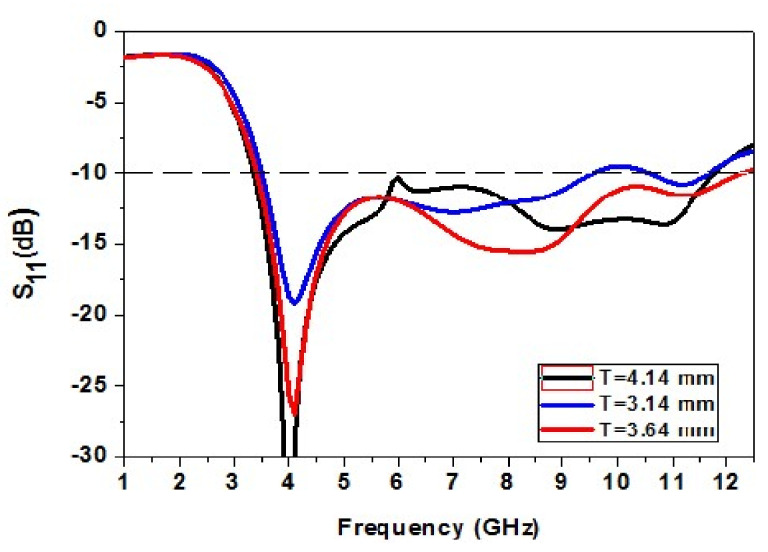
Analysis of change in the dimension T.

The size T of the ground plane is increased and decreased to analyze its effect. The increase in T causes a decrease in impedance bandwidth. The reduction in T also causes a fall in impedance bandwidth. The dimension T = 3.64 mm gives the correct bandwidth. So, T = 3.64 mm is an optimized value. Figure 5 shows the variation of the dimension T of the ground plane.

#### 2.2.3. Outcome of Feed Width W_f_ Variation

**Figure 6:**
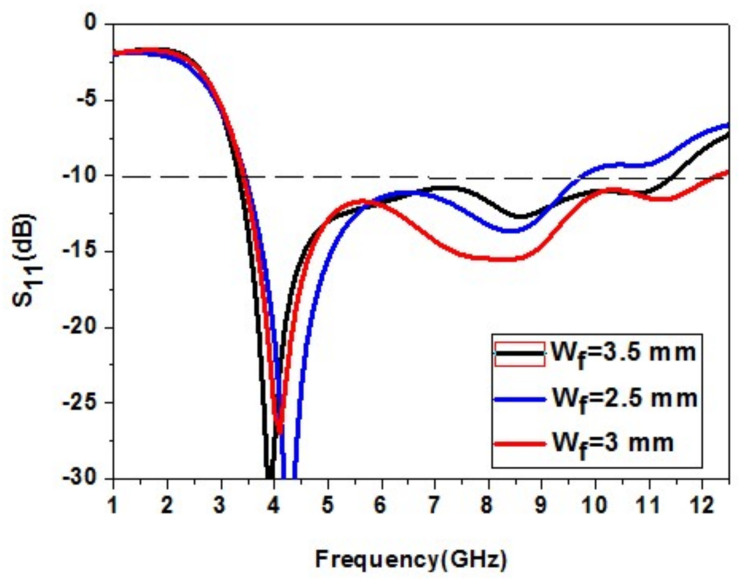
Analysis of the feed width W_f_ variation.

The effect of the feed width W_f_ of the patch element on the antenna is studied. The feed width W_f_ is increased and decreased to analyze its effect. The increase in W_f_ causes a decrease in impedance bandwidth. The reduction in W_f_ also causes a greater decrease in impedance bandwidth. The feed width W_f_ = 3 mm gives the correct bandwidth of 12.3 GHz. So, W_f_ = 3 mm is an optimized value. The variation of the feed width W_f_ of the patch element is shown in Figure 6.

#### 2.2.4. Outcome of Change in the Ground Plane Structure of UWB Antenna

**Figure 7:**
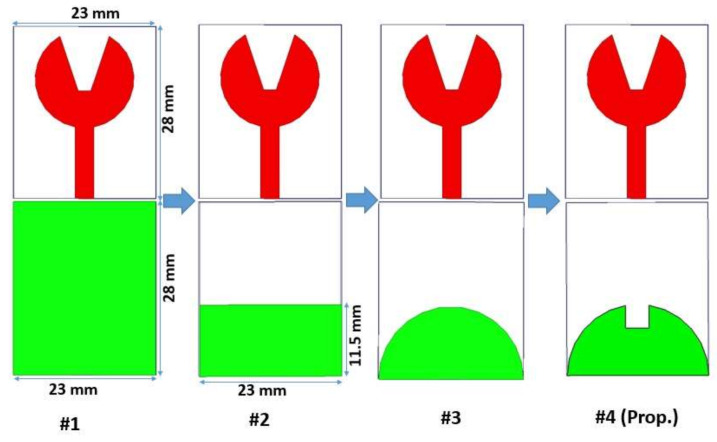
Variation of the ground plane structure.

Figure 7 shows the effect of variation of ground plane structure on the proposed design. In #1, the design has a full rectangular ground plane, which results in a narrow −10 dB impedance bandwidth from 8.4 to 10.2 GHz. To make a UWB, the impedance bandwidth is to be made wide, and truncation is done on the ground plane to make it a partial rectangular ground plane. This is shown in #2 of Figure 7. Compared to #1, the impedance bandwidth is 3.4–9.3 GHz, but it is not proper enough.

Therefore, further modification is done in #3 of Figure 7 to achieve UWB characteristics. The ground plane is further modified into a semicircular partial ground plane, which results in a narrow −10 dB impedance bandwidth from 3.5 to 7.8 GHz, which is not proper enough.

**Figure 8:**
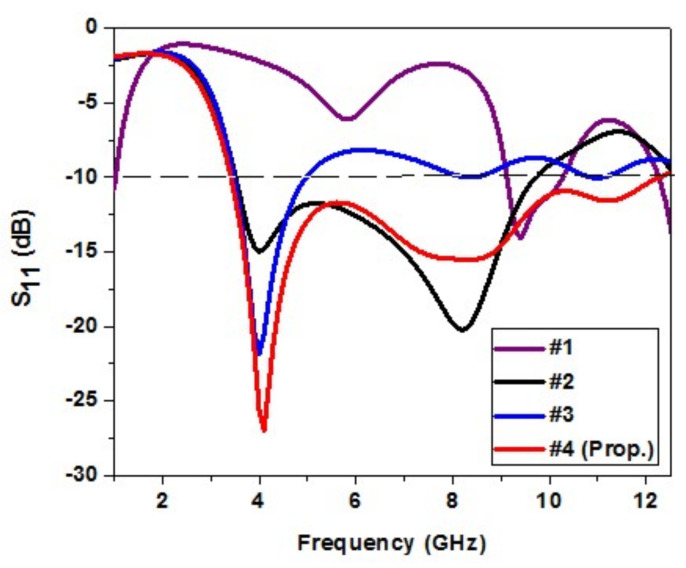
Analysis of variation of the ground plane structure.

A further modification is done in #4 of Figure 7 to achieve UWB characteristics in the antenna. A rectangular slot is cut into a semicircular partial ground plane. It results in an impedance bandwidth from 3.4 to 12.3 GHz, which covers the UWB spectrum (3.1–10.6 GHz). Figure 8 shows the variation in the ground plane structure.

### 2.3. Current Distribution of UWB Antenna

**Figure 9:**
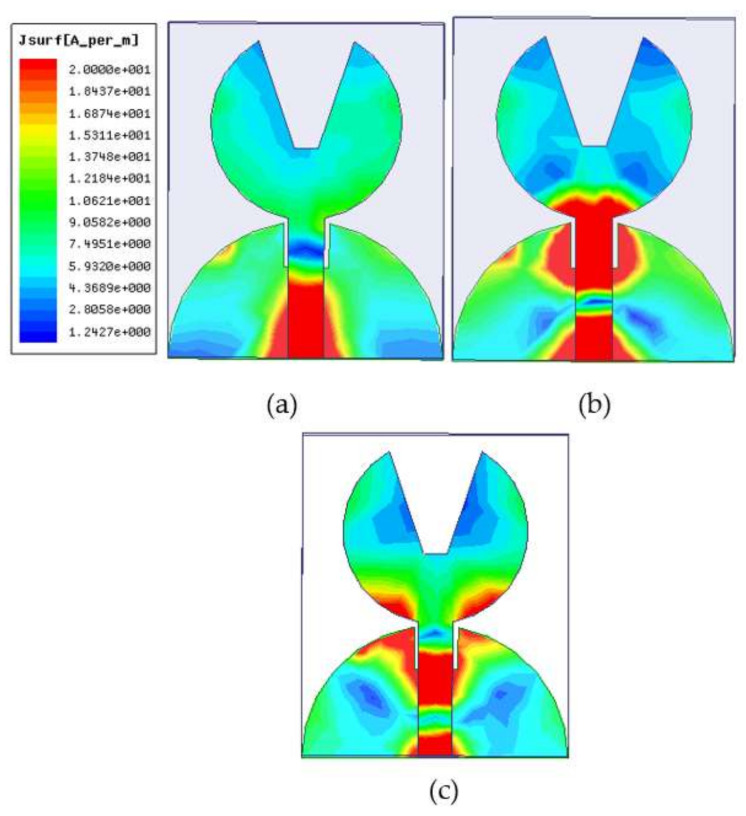
Current density plots for frequencies (**a**) 4.21 GHz and (**b**) 8.6 GHz and (**c**) 11.2 GHz.

The current density is shown in Figure 9a–c at 4.21, 8.61, and 11.2 GHz, respectively. The current pattern at the first, second, and third resonance frequencies of 4.1, 8.61, and 11.2 GHz depict first, second, and third harmonics, respectively. From the current distribution pattern, it can be stated that the majority of the currents are found around the edge of the radiating patch and the ground plane, while the currents at the center of the patch and ground plane are very weak. It is also seen that the current is coupled from the top and bottom edge of the ground plane to the patch through the microstrip feed line and radiates to the free space. The truncation on the top edge of the partial ground plane effectively alleviates the changes in antenna impedance by altering the current path and creating a symmetrical current distribution to a small ground plane, reducing the effect of the ground plane on the antenna’s performances. However, the currents are mainly distributed on the microstrip line and the junction between the patch and ground plane at higher frequencies. As a result, the currents on the ground plane become stronger than the lower frequencies, and impedance matching becomes worse for traveling wave-dependent modes.

## 3. UWB Notch Antenna Design Approach

**Figure 10:**
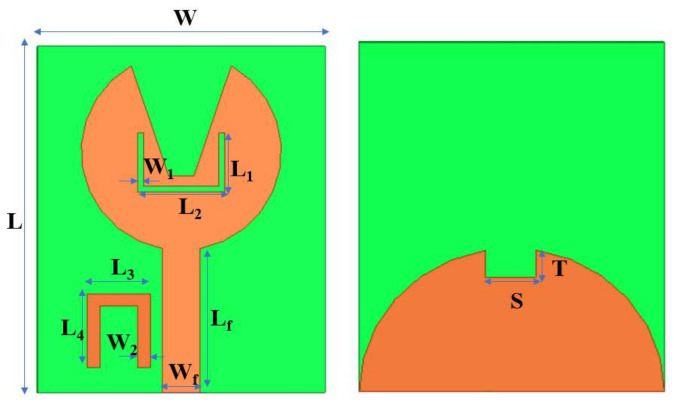
Designed UWB notch antenna.

Figure 10 represents the dual band-notched antenna’s geometry, with the top having V-shape slots and a U-shape truncated semicircular ground plane at the bottom.

**Figure 11:**
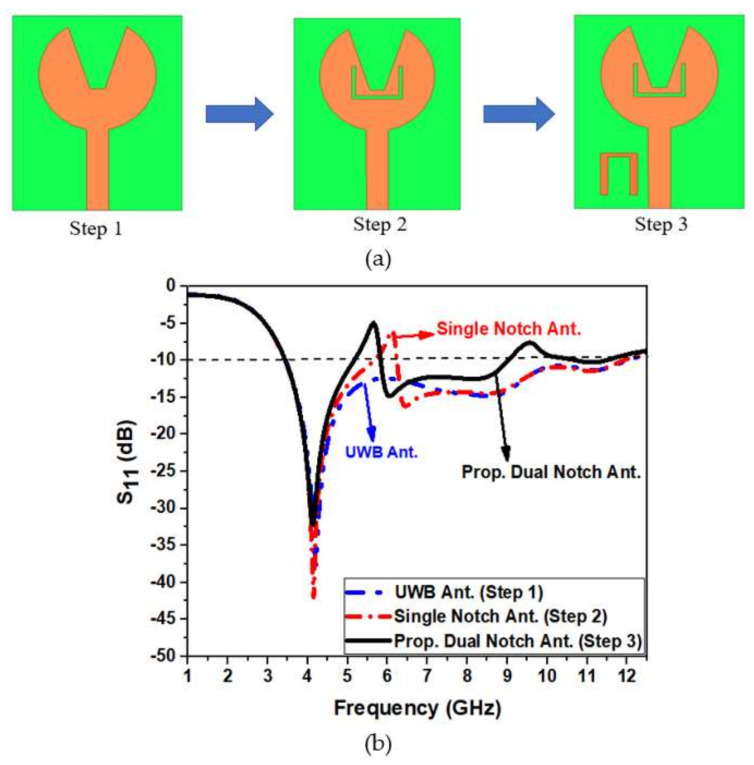
Implementation of (**a**) a dual notch in UWB antenna design and (**b**) corresponding S11 of the implementation stage.

A dual-band notch is acquired by cutting a U slot on the patch and embedding an inverted U-shape parasitic resonator near the feed line on the substrate’s top side. The U-shape slot is responsible for notching frequencies from 5.15 to 5.6 GHz, corresponding to the IEEE 802.11a WLAN system. The inverted U-shape parasitic resonator corresponds to the notching of frequencies from 9.13 to 10.5 GHz, which lies in the X-band. To obtain the dual notch characteristic from the UWB antenna, different shapes and techniques are employed with a series of steps involved. Finally, Figure 11a shows the U-shape slot and U-shape parasitic resonator selection. The corresponding S11 evolution of the notch antenna is illustrated in Figure 11b. The notch obtained is adjusted by altering the length of the slot and resonator, optimizing the parasitic resonator’s distance from the feed line. The slot resonates at the frequency when its length is half the wavelength. By adjusting its length and gap, destructive interference occurs at the desired notch frequency. The first notch frequency at 5.6 GHz is created with the help of a U-shaped slot, which can be mathematically determined by using Equation (4).

(4) $$\begin{array}{ll} f_{notch@5.6\;\mathrm{GHz}} & =\frac{c}{2\left(L_{1}+L_{2}+2W_{1}\right)\sqrt{\varepsilon_{eff}}}\\ & =\frac{3\times 10^{11}}{2\left\{4.8+7+\left(2\times 1\right)\right\}\sqrt{3.74}}=5.6\;\mathrm{GHz} \end{array}$$

where *c* represents the light’s speed, $\varepsilon_{eff}$ is the effective dielectric constant, *L*_1_ and *L*_2_ are the U-shaped slot length, and W_1_ is the U-shaped slot gap on radiating patch.

The second notch frequency at 9.6 GHz is created with the help of an inverted U-shaped parasitic resonator and can be mathematically determined by using Equation (5).

(5) $$\begin{array}{ll} f_{notch@9.6\;\mathrm{GHz}} & =\frac{c}{2\left(L_{3}+L_{4}-3W_{2}\right)\sqrt{\varepsilon_{eff}}}\\ & =\frac{3\times 10^{11}}{2\left\{5+6-\left(3\times 1\right)\right\}\sqrt{3.74}}=9.6\;\mathrm{GHz} \end{array}$$

where $L_{3}$ and $L_{4}$ are the inverted U-shaped parasitic resonator width and length, respectively, and $W_{2}$ is the resonator gap.

### 3.1. Parametric Analysis of UWB Notch Antenna

The X-band notch bandwidth is managed by adjusting the resonator’s length and the distance between the feed line and the resonator. The design parameters of the slot and resonator are L_4_ = 6 mm, L_3_ = 5 mm, L_2_ = 7 mm, L_1_ = 4.8 mm, W_1_ = 1 mm, and W_2_ = 1 mm. The desired narrow band of WLAN is sharpened by adjusting the length to about half the wavelength at the required frequency. The longer the resonator’s length, the lower the stopband frequency. Similarly, the smaller the distance between the feed line and parasitic resonator, the wider the stopband bandwidth gets, and vice versa for both cases. A parametric analysis of the slot etched and parasitic resonator embedded is being done for optimizing the antenna design.

#### 3.1.1. Outcome of Width W_1_ Variation of U-Shape Slot

**Figure 12:**
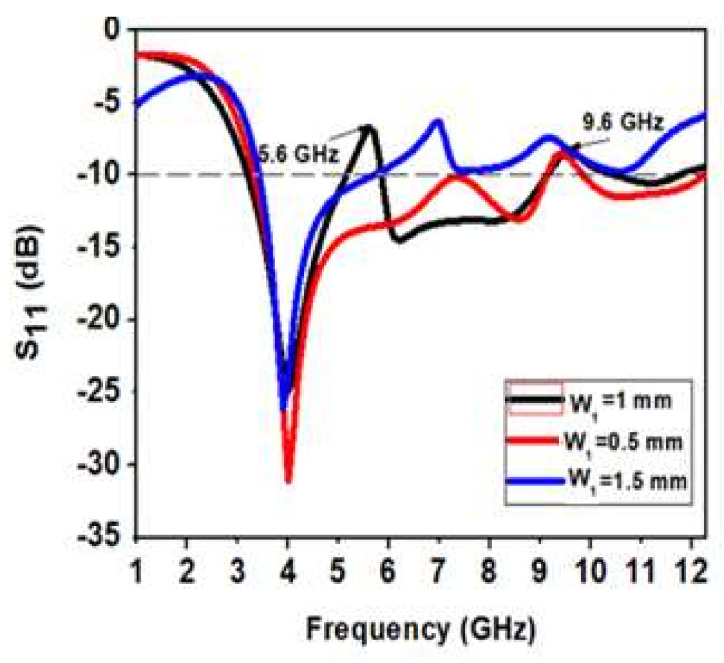
Analysis of variation in width W_1_ of the U-shape slot of the radiating patch.

Figure 12 shows the variation of width W_1_ of a U-shaped slot of the radiating patch. As W_1_ is decreased by 0.5 mm, only one band notch appears at 9.6 GHz. When W_1_ is increased by 0.5 mm, two notch bands are not proper, and the bandwidth is reduced. When W_1_ = 1 mm, there are two proper notch bands with a sharp rejection at 5.6 GHz and 9.6 GHz, respectively. Therefore, W_1_ = 1 mm is the most optimized value for a dual-band UWB notch antenna.

#### 3.1.2. Outcome of Width W2 Variation of Inverted U-Shape Parasitic Resonator

**Figure 13:**
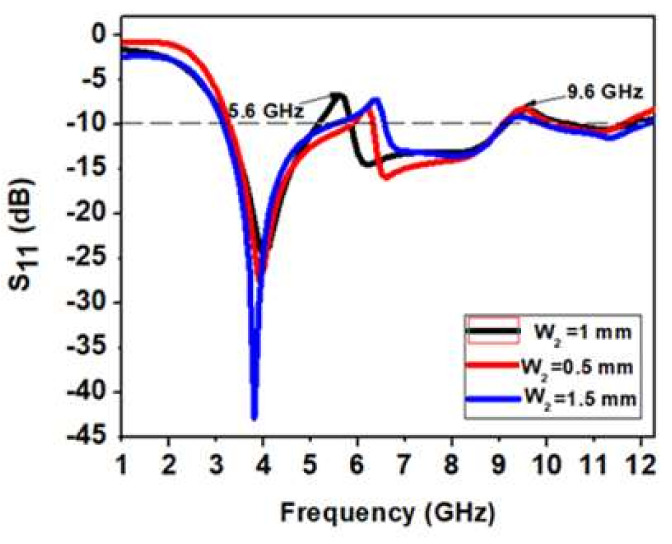
Analysis of variation in width W_2_ of an inverted U-shape parasitic resonator.

Figure 13 shows the variation of width W_2_ of the U-shaped parasitic resonator. As W_2_ is decreased by 0.5 mm, there is a shift in the notch band toward higher frequency, and the impedance bandwidth is reduced. When W_2_ is increased by 0.5 mm, there is a shift in the first notch band toward a higher frequency, and the second notch band to the −10 dB line. In addition, the impedance bandwidth is reduced. When W_2_ = 1 mm, there are two proper notch bands with a sharp rejection at 5.6 GHz and 9.6 GHz, respectively. Therefore, W_2_ = 1 mm is the most optimized value for a dual-band UWB notch antenna.

#### 3.1.3. Current Distribution of UWB Notch Antenna

**Figure 14:**
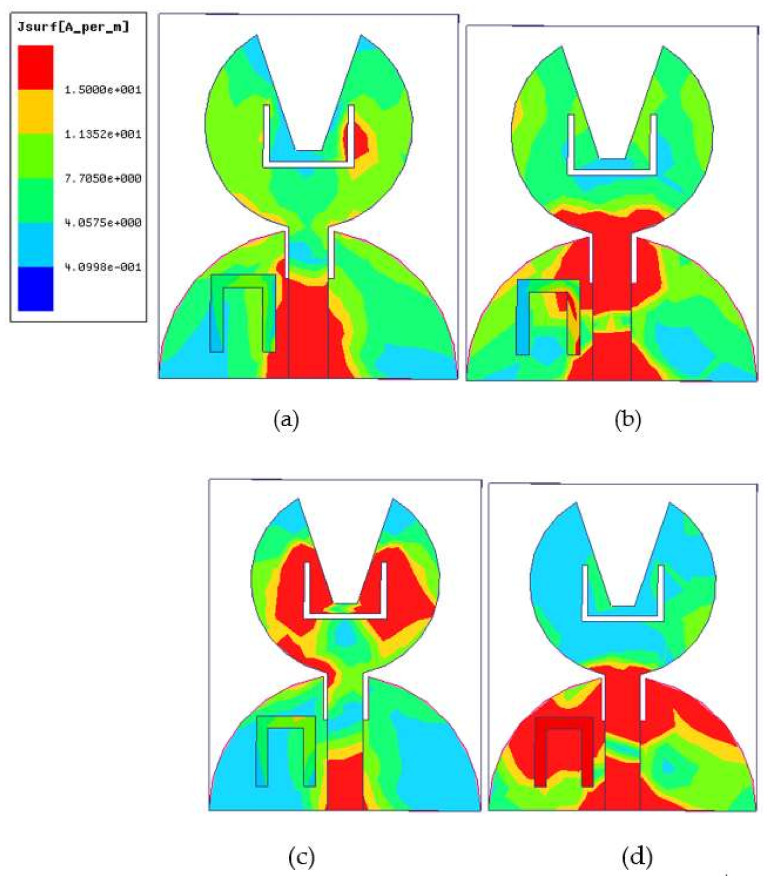
Current density plots at passband frequencies: (**a**) 4 GHz, (**b**) 6.2 GHz, and band-reject frequencies (**c**) 5.6 GHz and (**d**) 9.6 GHz.

Figure 14 shows the density of current of the designed UWB notch antenna at two passband frequencies (i.e., 4 and 6.2 GHz) and two-band notch frequencies (i.e., 5.6 and 9.6 GHz). At a resonant frequency of 4 and 6.2 GHz, the region colored in red in Figure 14a,b shows that the current density is highest in most of the radiation, while the current density is fairly uniform in other regions. The high current density is visible near the sharp edges of the slot, parasitic resonator, and feed line. At the WLAN band of Figure 14c, the current distribution is concentrated near the slot’s outer and inner edges in the patch at a band notch frequency of 5.6 GHz. Since the field generated near the slots is opposite in direction, they are responsible for notching the WLAN band’s frequency. Similarly, the current distribution is concentrated in the parasitic resonator responsible for the X-band frequency band. At 9.6 GHz (Figure 14d), the current density generated in the resonator due to magnetic flux generated in the feed line causes the induced current to flow in the resonator to neutralize the field generated in both the arms of the resonator. Therefore, the band-notched characteristic is obtained for the UWB notch antenna.

## 4. Results Obtained and Further Discussion

### 4.1. UWB Antenna Results

**Figure 15:**
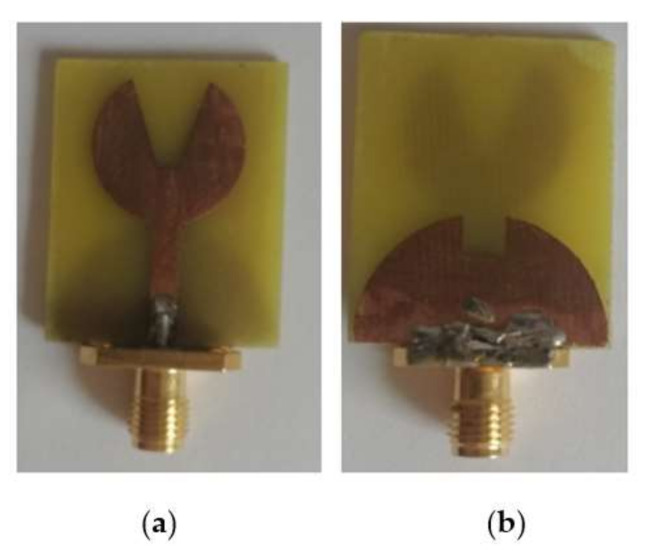
Fabricated proposed UWB antenna: (**a**) Top part (**b**) bottom part.

The proposed antenna has been simulated on an HFSSv.13.0 simulator using the FEM method and fabricated. Figure 15 shows the fabricated proposed UWB antenna.

#### 4.1.1. Simulated and Measured S_11_

**Figure 16:**
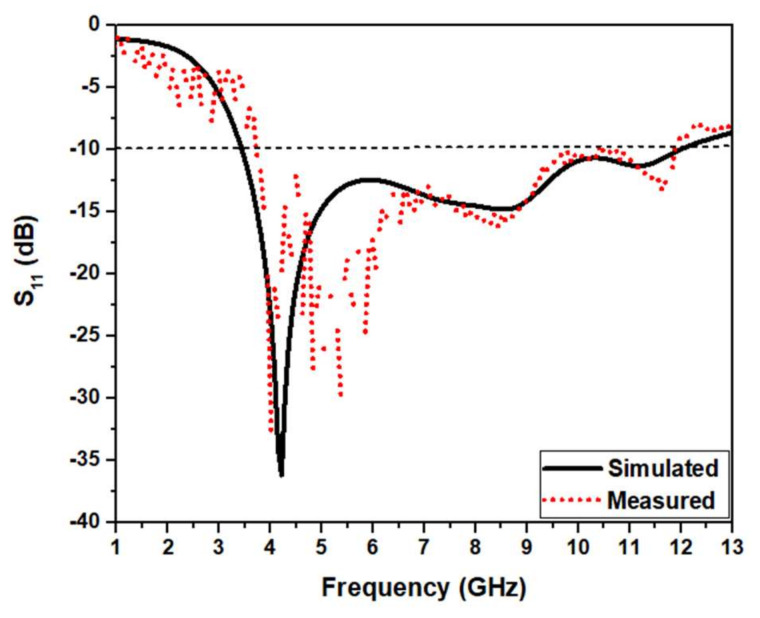
S_11_ parameter: simulated and measured.

Figure 16 shows the comparative analysis of the simulations and measurements of S11. S11 was measured using a ROHDE and SCHWARZ ZVL network analyzer. The simulated impedance bandwidth is 3.4–12.3 GHz at S11 < −10 dB level. The measured operating impedance bandwidth is 3.74–11.83 GHz at S11 < −10 dB level. There is a slight deviation between the simulations and measurements of S11. It is due to the effect of soldering, not considering the SMA connector during simulation, uncertainties in the substrate’s dielectric constant, and tolerances in fabrication.

#### 4.1.2. Radiation Pattern

**Figure 17:**
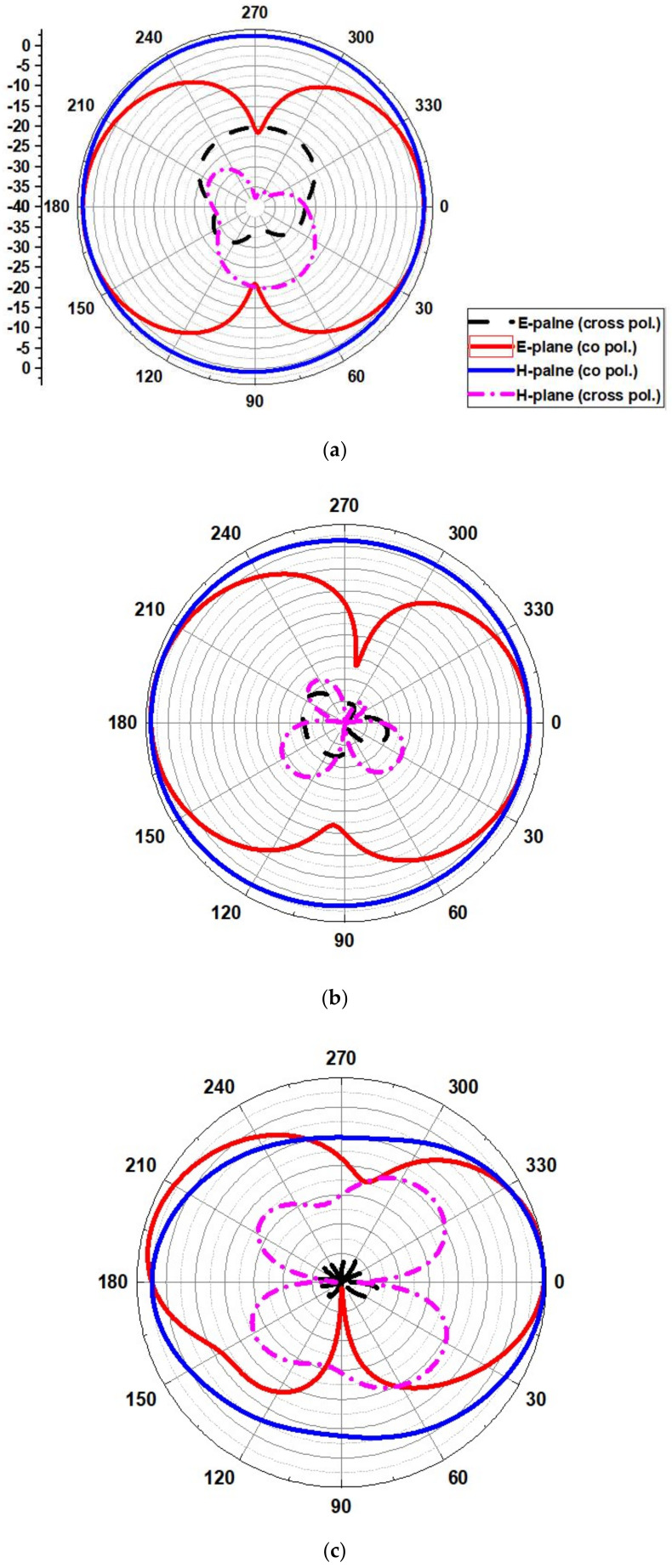

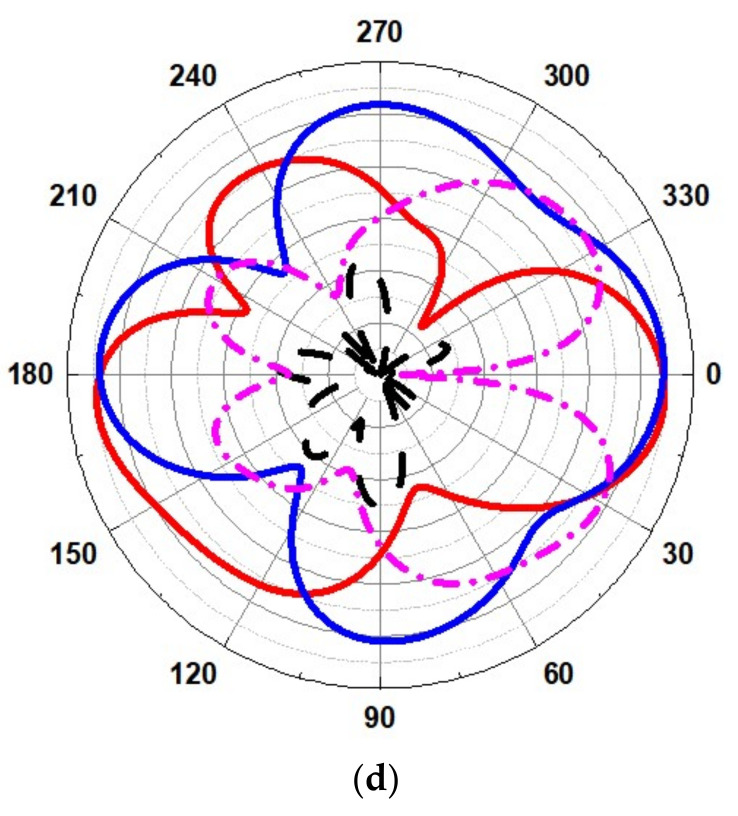
Radiation pattern at (**a**) 4.2 GHz, (**b**) 5.8 GHz, (**c**) 8.6 GHz, and (**d**) 11.2 GHz.

The radiation pattern obtained for operating frequencies of (4.2 GHz, 5.8 GHz, 8.6 GHz, and 11.2 GHz) for ∅ = 0° (H-plane) and ∅ = 90° (E-plane) is outlined in Figure 17. The simulated results show that the pattern of radiation in the H-plane is near omnidirectional, and in the E-plane, it is bidirectional and stable.

#### 4.1.3. Characteristics in the Time-Domain 

**Figure 18:**
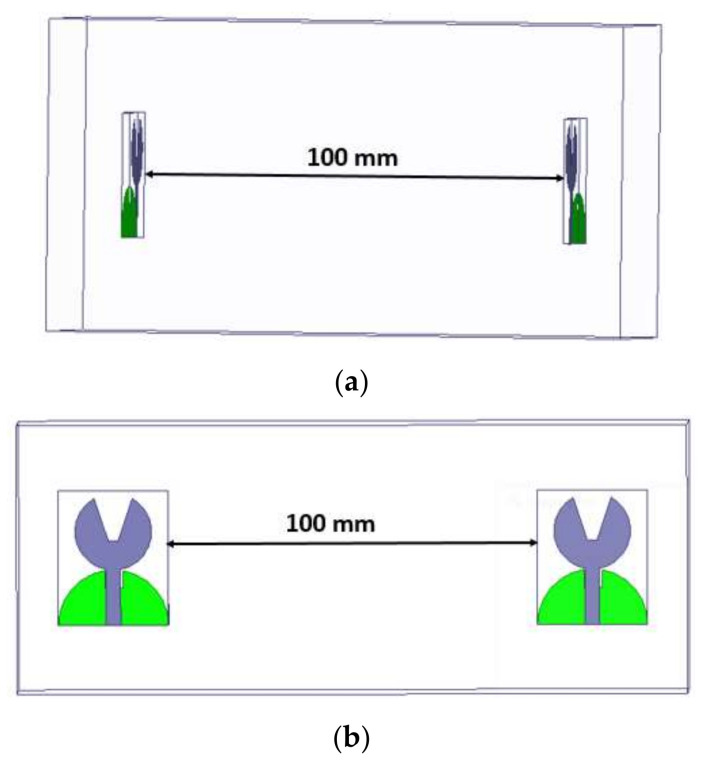
Time-domain analysis with orientations viz. (**a**) frontal and (**b**) lateral.

The analysis of the designed antenna in the time-domain viz., comprising of the phase response, group delay, and isolation characteristics, is performed. These studies have been performed by placing two antennas that are identical with a spacing of 100 mm in HFSS, as shown in Figure 18. Both lateral and frontal conditions have been used for performing the time-domain analysis.

**Figure 19:**
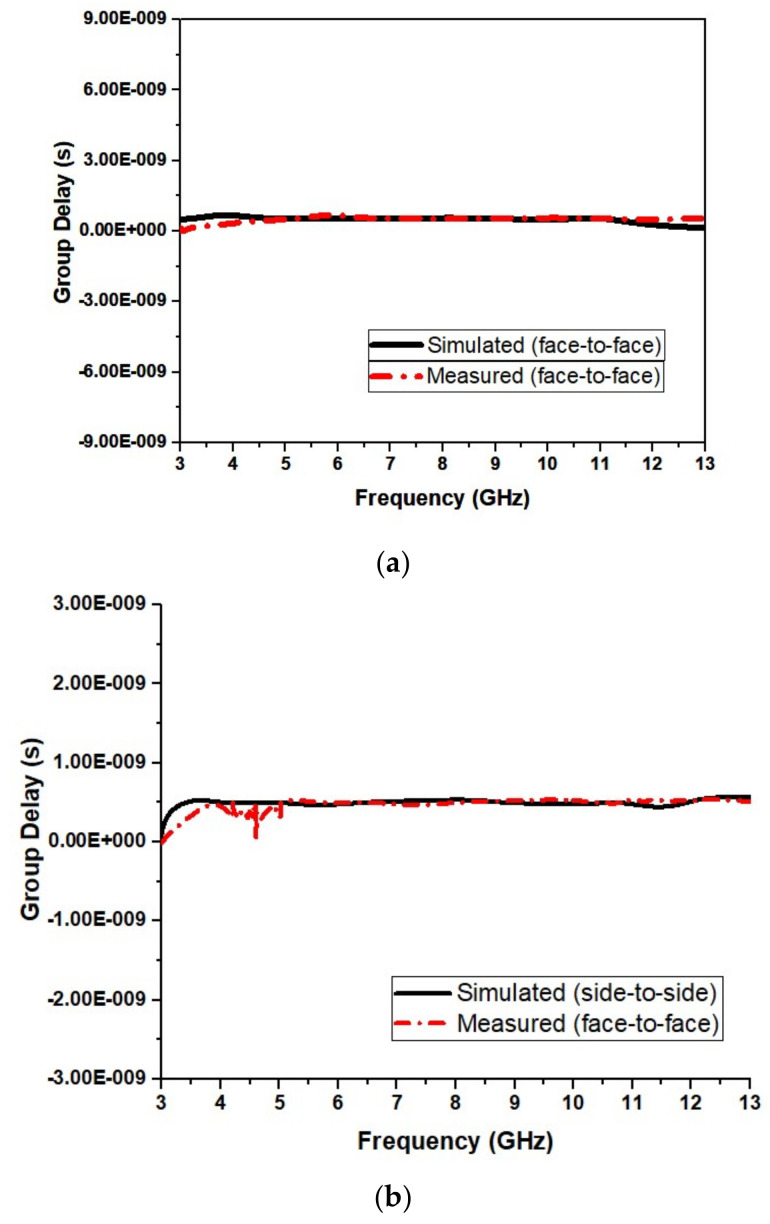
Group delay (**a**) frontal and (**b**) lateral positions of the proposed UWB antenna.

Figure 19 shows the group delay of the designed UWB antenna. As evident in Figure 19, group delay is almost linear for the antenna in both frontal and lateral conditions.

**Figure 20:**
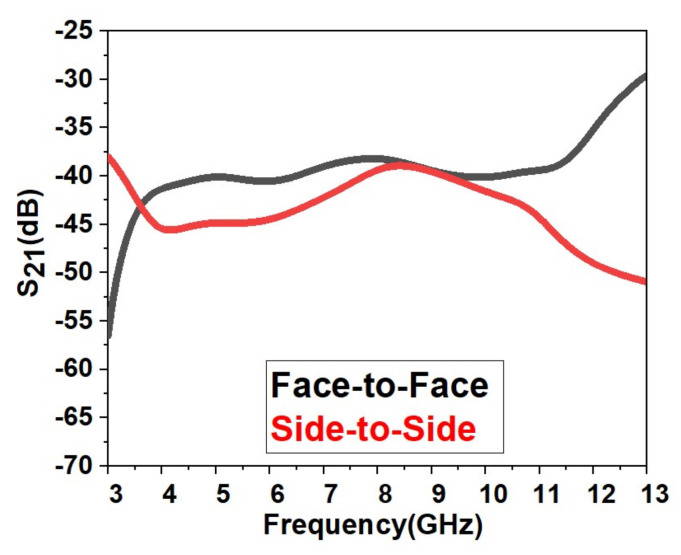
Isolation achieved for the design.

**Figure 21:**
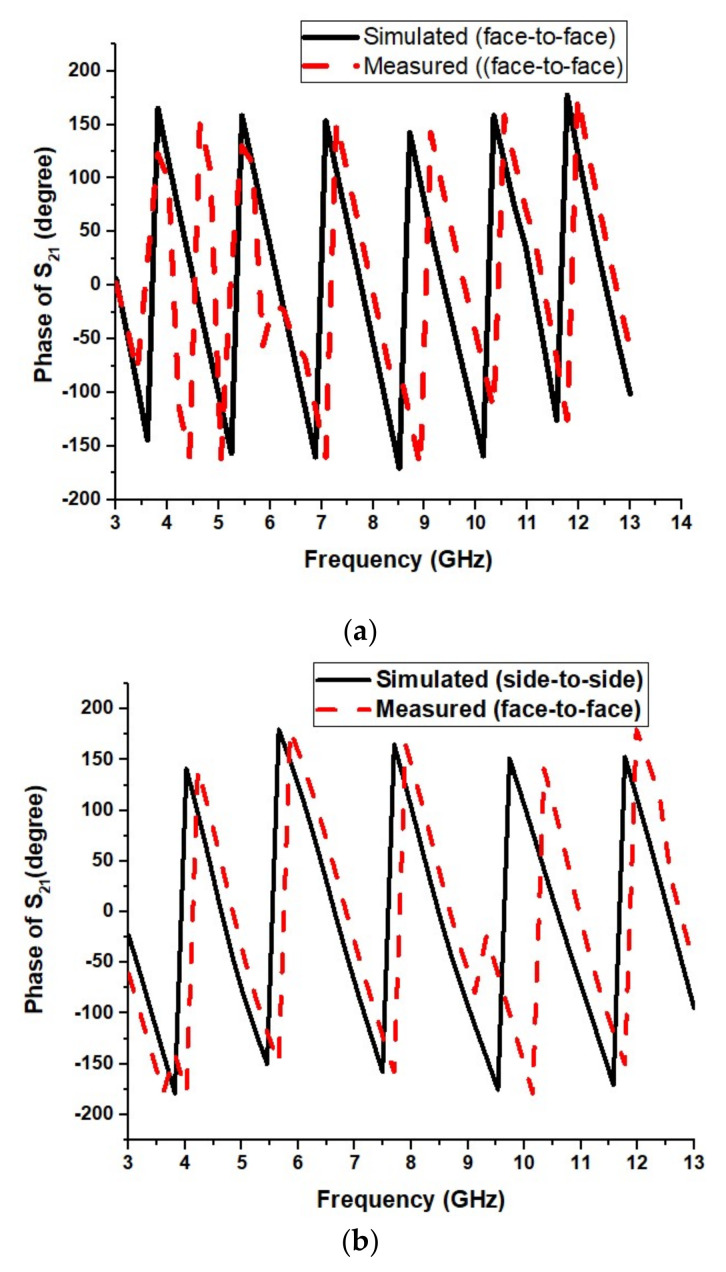
Phase response S_21_ of the proposed design: (**a**) frontal and (**b**) lateral.

Figure 20 shows the proposed UWB antenna’s isolation characteristics (S21). Figure 21 shows that acceptable isolation characteristics are obtained by the designed antenna (S21 < −20 dB) in frontal and lateral conditions.

Figure 21 shows the phase response of the designed UWB antenna. The phase should vary linearly for good time-domain characteristics. Figure 20 shows that the phase variation is almost linear for the antenna in frontal and lateral conditions.

**Figure 22:**
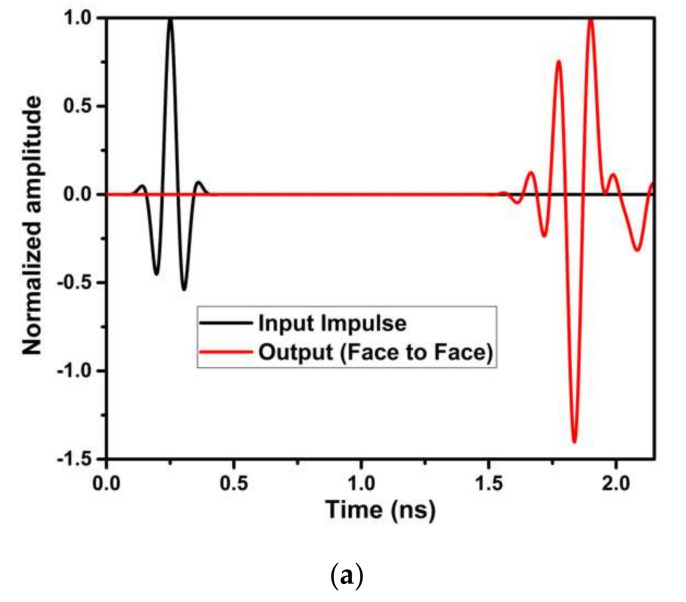

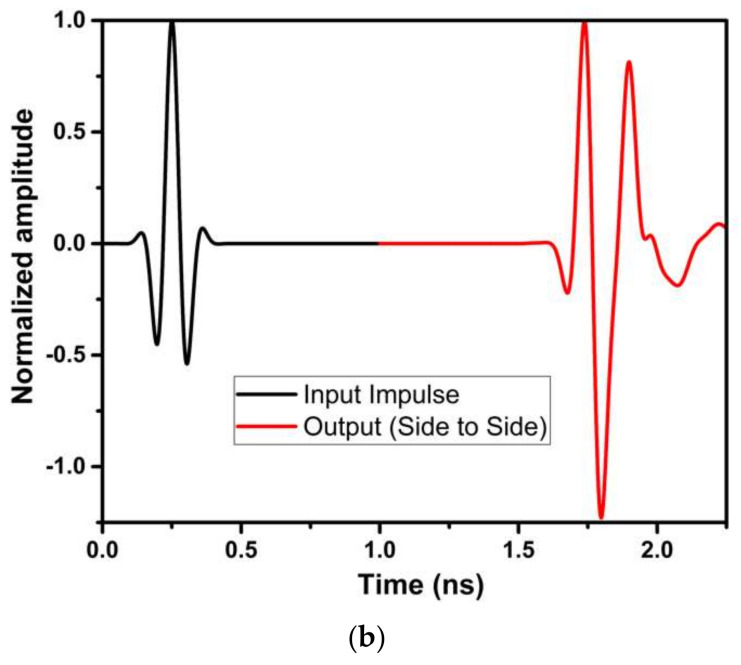
Fidelity Factor for (**a**) frontal (**b**) lateral conditions of the UWB antenna.

Figure 22 shows the proposed UWB antenna’s Fidelity Factor (FF) in frontal and lateral conditions. FF is 95.52% and 96.89% in frontal and lateral configuration. The higher value of FF ensures the similarity between the transmitted and received pulse.

#### 4.1.4. Gain of the UWB Antenna

**Figure 23:**
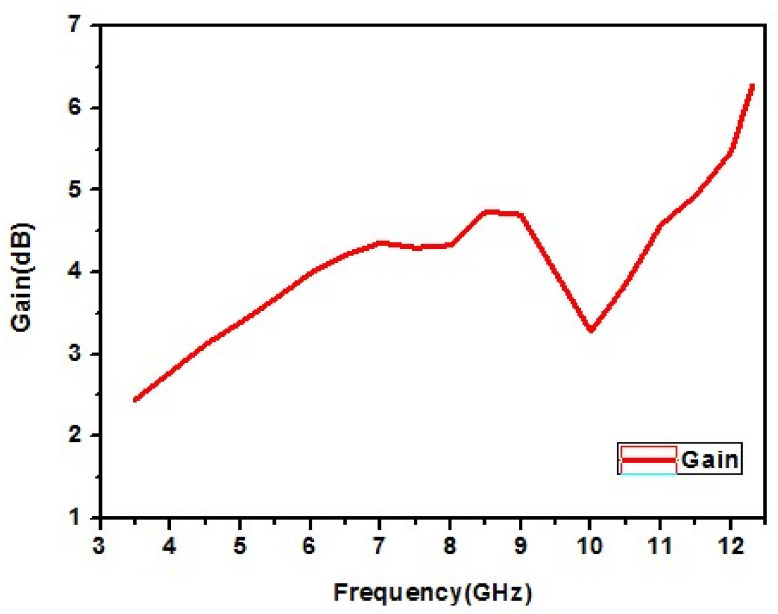
Gain of the designed UWB antenna.

Figure 23 shows that a maximum gain of 6.21 dB is generated at 12.3 GHz for the designed UWB antenna.

#### 4.1.5. Comparison Table

Table 2 compares the proposed design with the existing designs in the literature. It is noticeable from Table 2 that our design is superior in terms of performance when compared to the existing literature based on size, operation, and design complexity.

### 4.2. UWB Notch Antenna Results

**Figure 24:**
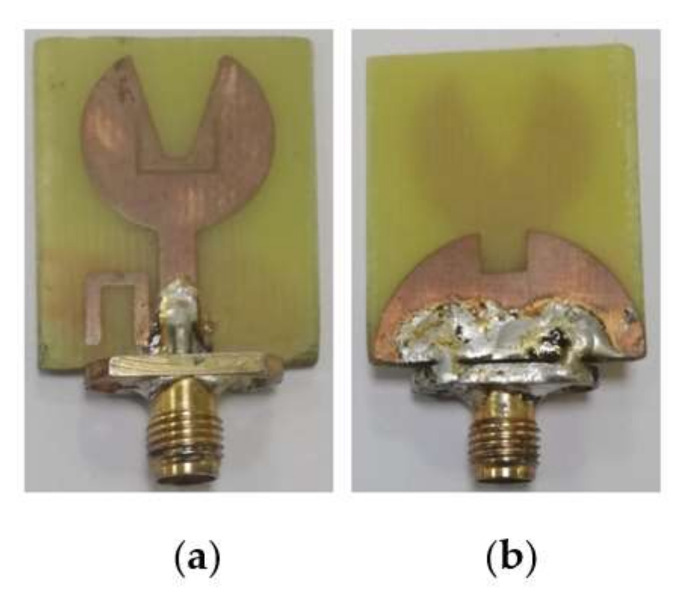
Fabricated proposed UWB notch antenna: (**a**) Top part (**b**) bottom part.

The designed UWB notch antenna is simulated on HFSSv.13.0 simulator. Figure 24 shows the fabricated proposed UWB notch antenna.

#### 4.2.1. Simulated and Measured S_11_

**Figure 25:**
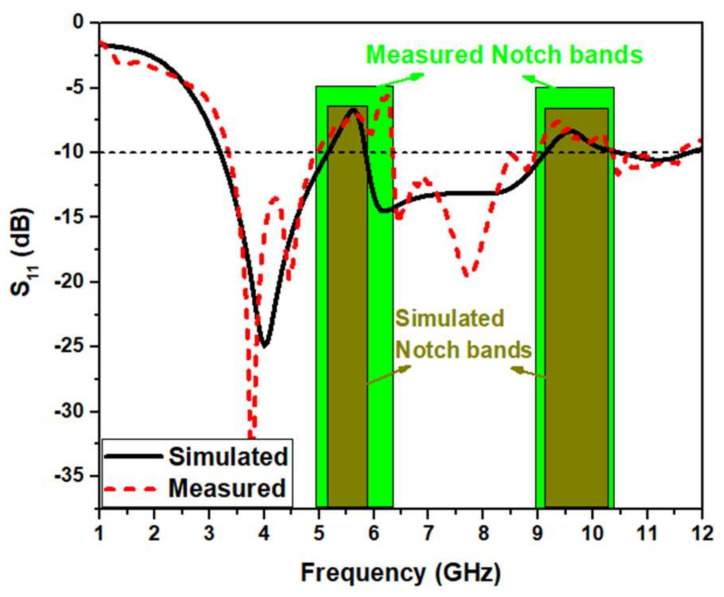
Simulations and measurements of S_11_ of the proposed UWB notch antenna.

Figure 25 compares the reflection coefficient of the simulations and measurements. Both values are almost similar in the UWB range. The simulated reflection coefficient plot shows strong rejection in the WLAN band from 5.15 to 5.8 GHz with a sharp notch at 5.6 GHz and X band from 9.1 to 10.5 GHz with a sharp notch at 9.6 GHz, having a fractional bandwidth of about 114 % (3.2–11.7 GHz). The measured reflection coefficient plot shows strong rejection in the WLAN band from 5.0 to 6.3 GHz with a sharp notch at 6.25 GHz and X band from 9.0 to 10.35 GHz with a sharp notch at 9.4 GHz, having a fractional bandwidth of 110% (3.3 GHz to 11.5 GHz). The simulations and measurements of S11 are similar with a slight deviation. It may be owing to the soldering effect, not considering the SMA connector for simulation, uncertainties in the substrate’s dielectric constant, and tolerances in fabrication.

#### 4.2.2. Radiation Pattern

**Figure 26:**
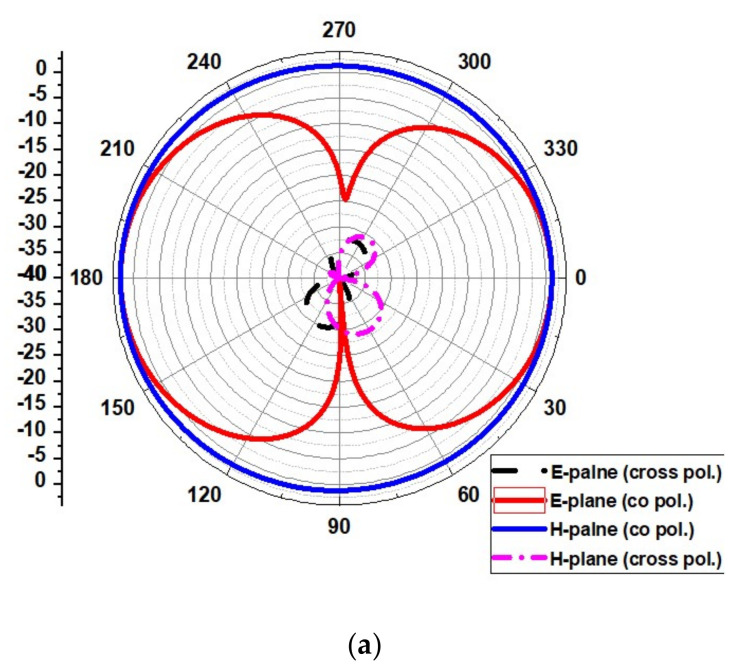

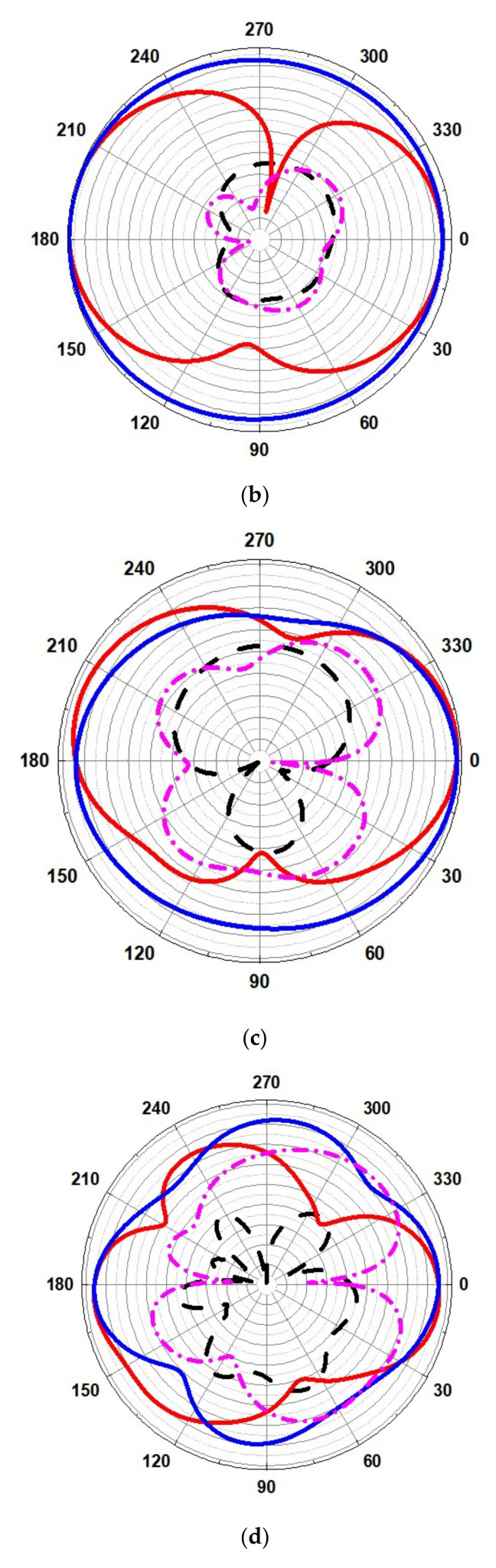
Radiation pattern of the proposed UWB notch antenna at (**a**) 4 GHz, (**b**) 6.2 GHz, (**c**) 8.4 GHz, and (**d**) 11.2 GHz.

Figure 26 shows the UWB notch antenna’s radiation pattern at the resonance frequency of 4, 6.2, 8.4, and 11.2 GHz.

#### 4.2.3. Time-Domain Characteristics

**Figure 27:**
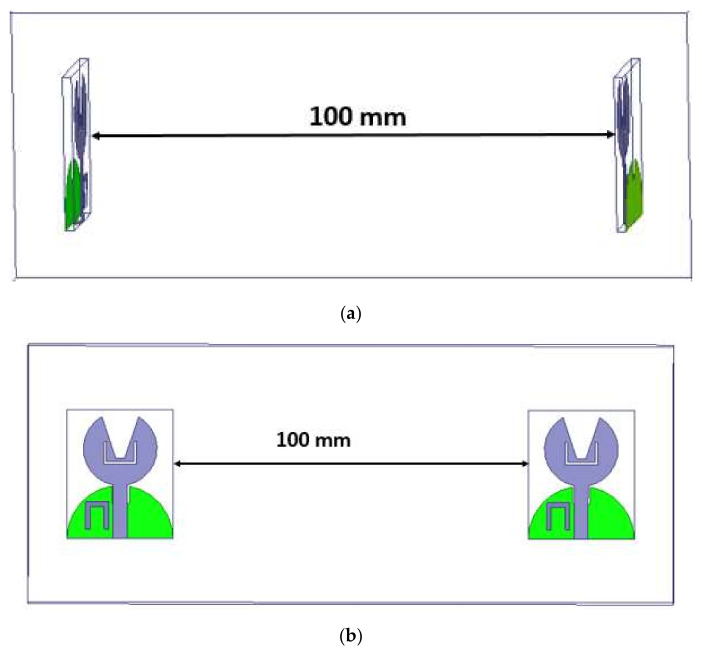
Time-domain analysis with orientations viz. (**a**) frontal and (**b**) lateral.

The time-domain analysis, i.e., phase response and group delay for the proposed antenna, are performed as shown in Figure 27. These studies are performed in the same manner as described in Section 4.1.3.

**Figure 28:**
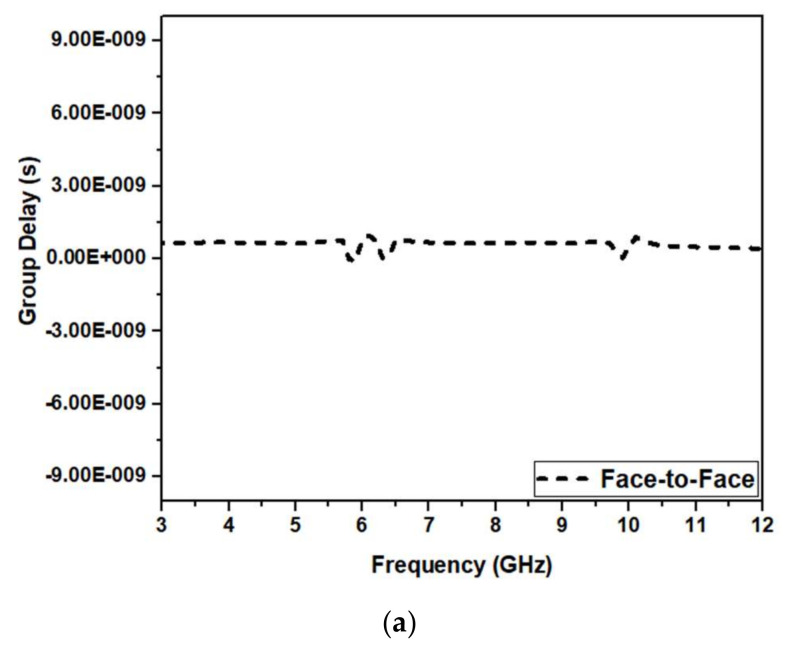

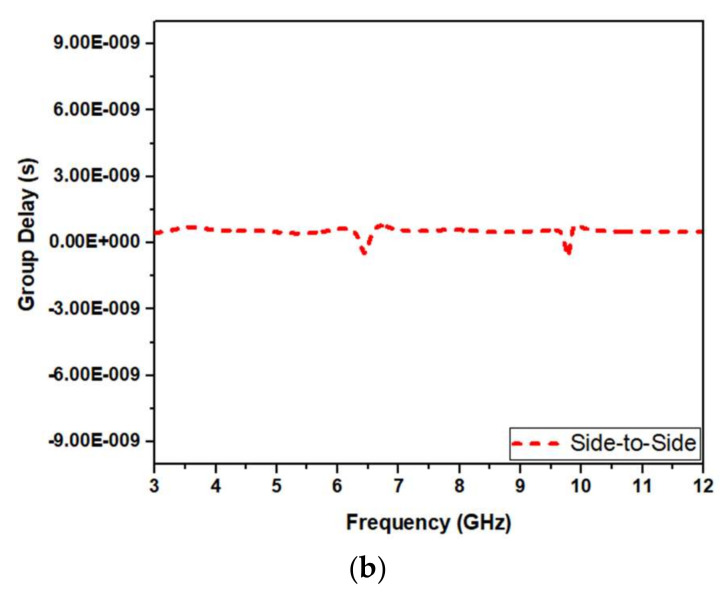
Group delay (**a**) frontal and (**b**) lateral orientations of the proposed UWB notch antenna.

The group delay for our design in frontal and lateral orientations is shown in Figure 28a,b, respectively. Conventionally, the group delay should be constant for the frequency of operation except for the notches. Figure 28a,b show that values for group delay are <1 ns and constant except at the notched bands.

**Figure 29:**
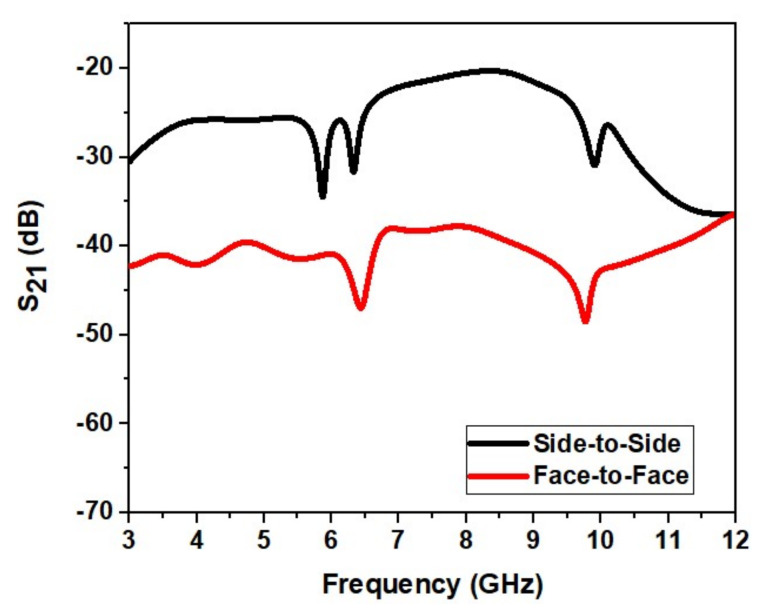
Isolation achieved for the UWB notch antenna.

Figure 29 shows the proposed UWB notch antenna’s isolation characteristics (S21). Figure 29 shows that acceptable isolation characteristics are obtained by the designed antenna (S21 < −25 dB) in frontal and lateral conditions.

**Figure 30:**
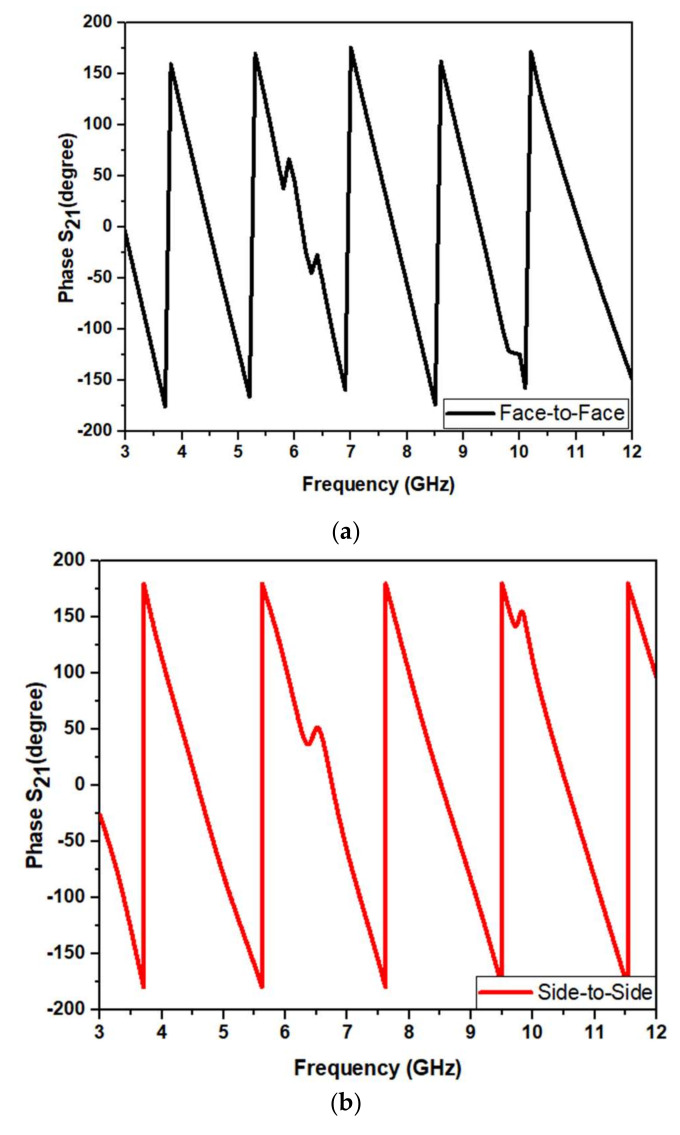
Phase response S21 of the proposed UWB notch antenna (**a**) frontal (**b**) lateral configurations.

Figure 30a,b show the proposed UWB notch antenna’s phase response. Conventionally, the phase response should vary linearly for the operational frequency band and not in the notched bands. It is observed from the above figure that the phase response for both frontal and lateral configuration are almost linear.

**Figure 31:**
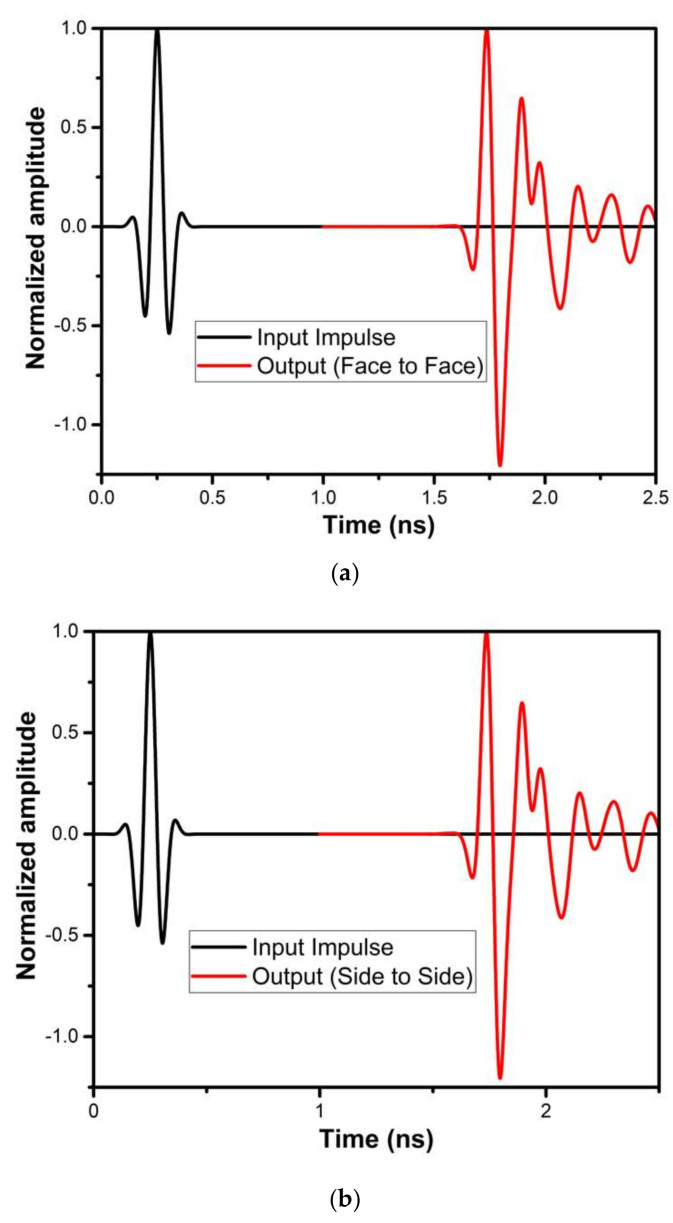
Fidelity Factor for (**a**) frontal (**b**) lateral conditions of UWB notch antenna.

Figure 31 shows the proposed UWB notch antenna’s Fidelity Factor (FF) in frontal and lateral conditions. The obtained FF are 94.47% and 95.58% in frontal and lateral configuration, respectively. The higher value of FF ensures the similarity between transmitted and received pulses.

#### 4.2.4. Comparison Table

Table 3 compares our design with the existing UWB notch antenna in the literature. Our design outperforms the designs in the existing literature in terms of size, operation, and design complexity.

## 5. Conclusions

A V-shaped UWB monopole antenna and dual-band UWB notch antenna are simulated, fabricated, and measured. A rectangular slot is cut into a semicircular partial ground plane of the UWB antenna and radiates in the UWB spectrum. The dual-band notch is obtained by cutting a U-shaped slot on the patch of radiation and embedding an inverted U-shape parasitic resonator on top of the substrate near the feed line. Our design showcases an impedance bandwidth of 3.4 to 12.3 GHz, group delay < 1 ns, and a stable radiation pattern. The proposed UWB notch antenna has an impedance bandwidth of 3.2–11.7 GHz and dual-band notches at 5.15 to 5.8 GHz and 9.1 to 10.5 GHz. The UWB notch antenna has group delay < 1 ns and linear phase response in the frequency of operation except at the notched frequency bands. The proposed UWB and UWB notch antennas are compared with the existing literature. Less group delay, linear phase, stable radiation patterns, and dual notch bands make it feasible for usage in UWB applications.

## Figures and Tables

**Table 1:** Dimensions of proposed UWB antenna design.

Parameter	Dimensions (mm)
Substrate height, h	1.6
Substrate width, W	23
Substrate length, L	28
Perimeter of the patch	36.71
L_f_	11.64
W_f_	3
X	5.9
Y	9.42
Z	2
S	3.8
T	3.64

**Table 2:** Comparison of the proposed UWB antenna with the existing antenna in the literature.

Ref.	Electrical Size ($\lambda_{0}$ is Calculated at the First Resonance)	Range (GHz)	Gain (dB)	Operating Band (GHz)	Radiating Patch Configuration	Complexity	Ground Plane Structure	Substrate
[34]	$0.40\lambda_{0}\times 0.23\lambda_{0}$	3–11	4.16	3.5, 6.7, 9.8	Elliptical MSA juxtaposed with the ground pattern	No	Partial ground plane with rectangular notch	FR4 epoxy, h = 0.8 mm, *ε*_r_ = 4.4
[35]	$0.4\lambda_{0}\times 0.5\lambda_{0}$	2.85–15	2.79	3, 6, 8.7, 12	Planar multilateral disc sharp radiator	No	Rectangular ground plane with feed gap T	FR4 epoxy, h = 1.6 mm, *ε*_r_ = 4.4
[36]	$0.52\lambda_{0}\times 0.43\lambda_{0}$	4–11	4.65	5.2, 8	CPW-fed step-shaped radiating element	Yes	Defective ground plane	FR4 epoxy, h = 1.6 mm, *ε*_r_ = 4.4
[37]	$0.53\lambda_{0}\times 0.53\lambda_{0}$	2.8–11	3.1	3.2, 7.3	Coplanar waveguide (CPW)-fed UWB printed antenna	Yes	Closed ring resonators (CRRs) on a ground plane	Taconic, h = 1.575 mm, *ε*_r_ = 2.33, tan *δ* = 0.0009
[38]	$0.58\lambda_{0}\times 0.40\lambda_{0}$	3–12	4.2–6.8	5, 9.2	Ring-shaped with additional slot	No	Partial ground consisting of slot in rectangular shape on the top	FR4 epoxy, h = 1.6 mm, *ε*_r_ = 4.4, tan *δ* = 0.02
[39]	$0.57\lambda_{0}\times 0.32\lambda_{0}$	3.1–16.8	5	4.9, 10, 14.2, 16	Quarter circular patch with tapered microstrip feed line	No	Partial	FR4 epoxy, h = 1.6 mm, *ε*_r_ = 4.4, tan *δ* = 0.02
[40]	$0.44\lambda_{0}\times 0.44\lambda_{0}$	1.5–12	5.8	2, 4.8, 6.9, 9, 10.5	Printed pentagon monopole antenna	Yes	Partial	FR4 epoxy, h = 1.59 mm, *ε*_r_ = 4.4, tan *δ* = 0.01
[41]	$0.23\lambda_{0}\times 0.17\lambda_{0}$	2.37–12	4.95	1.5, 2.8, 4.9, 6.8, 7.9, 9.8, 11.2	Printed planar monopole antenna	Yes	Partial	Rogers 4003 C, h = 1.524 mm, *ε*_r_ = 3.55, tan *δ* = 0.0014
[42]	$0.39\lambda_{0}\times 0.34\lambda_{0}$	2.6–13.6	5.03	3, 4.5, 6.8	Microstrip-fed antenna in the shape of a flower	Yes	Semi-elliptical partial ground plane	FR4 epoxy, h = 1.6 mm, *ε*_r_ = 4.4, tan *δ* = 0.02
Proposed	$0.37\lambda_{0}\times 0.30\lambda_{0}$	3.4–12.3	6.21	4, 8.7, 11.8	V-shaped patch antenna	No	Semicircular partial ground plane	FR4 epoxy, h = 1.6 mm, *ε*_r_ = 4.4, tan *δ* = 0.02

**Table 3:** Comparison of proposed UWB notch antenna with the existing antenna in the literature.

Ref.	Electrical Size ($\lambda_{0}$ is Calculated at the First Resonance)	Range (GHz)	Technique	Complexity	Ground Plane Structure	Substrate
[43]	$0.32\lambda_{0}\times 0.3\lambda_{0}$	2.76–11	Quad stub-loaded offset annular ring structure	Yes	Two CSRR-loaded DGS	FR4 epoxy, h = 1.6 mm, *ε*_*r*_ = 4.4, tan *δ* = 0.024
[44]	$0.42\lambda_{0}\times 0.32\lambda_{0}$	2.7–14.9	Slot-type split-ring resonator (ST-SRR	Yes	Partial defected ground plane	FR4 epoxy, h = 1.6 mm, *ε*_*r*_ = 4.4, tan *δ* = 0.02
[45]	$0.20\lambda_{0}\times 0.26\lambda_{0}$	2–10.6	N-shaped stub inside the radiation path	Yes	Q-shaped stub on the ground	FR4 epoxy, h = 1 mm, *ε*_*r*_ = 4.5, tan *δ* = 0.0035
[46]	$0.25\lambda_{0}\times 0.25\lambda_{0}$	2.39–11.4	Quasi U-shape patch and four stub	Yes	Stepped slot on ground plane	FR4 epoxy, h = 1.6 mm, *ε*_*r*_ = 4.4, tan *δ* = 0.02
[47]	$0.26\lambda_{0}\times 0.40\lambda_{0}$	3–11	T-shaped slots in the U- shaped radiation patch	No	Complementary split-ring resonators (CSRRs) on both sides of the microstrip line	FR4 epoxy, h = 1.5 mm, *ε*_*r*_ = 4.4, tan *δ* = 0.02
Proposed	$0.37\lambda_{0}\times 0.30\lambda_{0}$	3.2–11.7	U-shaped slot on radiating patch with inverted U-shape parasitic resonator on the substrate	No	Semicircular partial ground plane	FR4 epoxy, h = 1.6 mm, *ε*_r_ = 4.4, tan *δ* = 0.02

## Data Availability

Not applicable.
